# The Impact of Postoperative Intravenous Iron Therapy on Clinical Outcomes in Surgical Patients with Iron-Deficiency Anemia: A Comparative Analysis by Frailty Status in the Setting of Elective Cardiac Surgery

**DOI:** 10.3390/medicina61111919

**Published:** 2025-10-26

**Authors:** Laser Şanal, Erdal Şimşek, Serdar Günaydın

**Affiliations:** 1 Transfusion Center, Ministry of Health, Ankara Bilkent City Hospital, Ankara 06800, Turkey; 2 Department of Cardiovascular Surgery, University of Health Sciences, Ankara Bilkent City Hospital, Ankara 06800, Turkey; erdal.simsek@sbu.edu.tr (E.Ş.); serdarkvc@gmail.com (S.G.)

**Keywords:** iron-deficiency anemia, frailty, elective cardiac surgery, intravenous iron therapy, postoperative, clinical outcomes

## Abstract

*Background and Objectives:* This study aimed to comparatively investigate the impact of postoperative intravenous iron therapy (IVIT) as an add-on to preoperative IVIT on clinical outcomes in frail versus non-frail patients with iron-deficiency anemia (IDA) in the setting of elective cardiac surgery. *Materials and Methods:* This was a retrospective single-center study. The data was collected prospectively between January 2021 and November 2024. A total of 200 patients with IDA (100 frail and 100 propensity-score-matched non-frail patients) who received IVIT before and/or after elective cardiac surgery were included. Patients were divided into four equal groups including frail pre/post group (frail patients with preoperative plus postoperative IVIT), non-frail pre/post group (non-frail patients with preoperative plus postoperative IVIT), frail pre group (frail patients with preoperative IVIT) and non-frail pre group (non-frail patients with preoperative IVIT). Perioperative parameters, postoperative complications, and length of hospital stay (LOS) were recorded in each group. Postoperative follow-up parameters included change in hemoglobin levels and reticulocyte count from baseline (on operation day, postoperative day 1, day 7, 1st month and 3rd month) as well as the hospital readmission and mortality rates within 3 months after surgery. *Results:* Hemoglobin levels (10.6 ± 1.2 g/dL at baseline to 12.6 ± 1.4 g/dL at 1st month and 13.4 ± 1.4 g/dL at 3rd month, *p* = 0.01 and *p* = 0.02) and reticulocyte counts (0.035 ± 0.005 × 10^12^/L at baseline to 0.075 ± 0.005 × 10^12^/L at 1st month and 0.085 ± 0.005 × 10^12^/L at 3rd month, *p* = 0.004 and *p* = 0.002) were significantly improved from baseline only in the frail pre/post group. *Conclusions:* Postoperative IVIT demonstrated improved postoperative hemoglobin levels and reticulocyte counts, besides its potential in reducing perioperative transfusions, in the setting of elective cardiac surgery in frail patients with IDA.

## 1. Introduction

Iron is an essential element of enzymes, cytochromes, and oxygen-carrying molecules (hemoglobin and myoglobin), due to its ability to donate and transport electrons as it changes from the trivalent ferric form to the bivalent ferrous form [1]. Among various types of anemia, iron-deficiency anemia (IDA) is particularly common, affecting up to 50% of patients undergoing major surgery [2]. Patients scheduled for cardiac surgery often have anemia and iron deficiency [3]. Iron-deficiency anemia (IDA) is frequent in patients undergoing cardiac surgery and is associated with increased morbidity and mortality [1,2,3,4]. IDA is further exacerbated for several weeks to months after the surgery due to significant blood loss in most cardiac surgical interventions, antiplatelet or anticoagulant medications, widespread use of agents that reduce gastric acidity, inflammation-induced worsening of iron sequestration, and increased erythropoiesis demand caused by pre-existing acute-on-chronic anemia, and thus, postoperative anemia occurs in 80–90% of cardiac surgery patients [5,6,7,8]. One of the strategies to manage such perioperative anemia is allogeneic blood transfusion and restoration of hemoglobin levels, but transfusion has multiple associated complications including alloimmunization, transfusion-transmitted diseases, transfusion reactions, etc. Blood is also a limited and costly resource. So, in the last few decades, the focus has shifted from blood transfusion to the importance of diagnosing, treating and preventing the underlying causes of anemia [9].

Blood transfusion practices, when considering their possible risks and side effects, have become more restrictive over the years with the emergence of the concept of Patient Blood Management (PBM) programs. PBM, defined as ‘a patient-centered approach to optimize patients’ endogenous red cell mass, to minimize blood loss in patients undergoing surgery, and to harness and optimize patient-specific physiological tolerance to anemia’, has been used at the national and institutional levels since the beginning of the millennium. Through such interventions, reduced transfusion of blood products or even ‘bloodless’ surgical care can be achieved, leading to fewer complications, less morbidity and mortality, and improved patient outcomes [8,10,11]. Unnecessary blood transfusions are of major concern, not only because of the negative impact on the health status of patients but also on the spending of healthcare budgets [11].

The impact of PBM in cardiac surgery patients has been explored in a number of studies with different perspectives and aims. These studies have concluded that there were statistically meaningful reductions in the number of RBC transfusions, morbidity, length of stay in hospital, and costs after implementation of a PBM program [11,12,13,14,15].

Postoperative anemia is a multifactorial condition, with iron deficiency being evident in the vast majority of anemic patients [7,8,16]. Oral iron supplements are often not effective in surgical patients as they are poorly tolerated because of side effects and have low bioavailability (10–20% absorption rate) which means that weeks to months of therapy are required to replenish iron stores [5]. Moreover, residual iron supplement remains largely unabsorbed in the gastrointestinal tract in post-surgical patients, leading to gastrointestinal side effects [8]. As an alternative, intravenous iron therapy (IVIT) is considered a favorable option to replenish iron stores in surgical patients, which can safely deliver ≥1000 mg of elemental iron in a single infusion. It is also better tolerated and more efficient for replenishing iron stores than oral iron [5,8,17,18].

Although use of IVIT in the preoperative period has yielded promising results in terms of improved hemoglobin concentrations (by 5–10 g/L) and reduced allogeneic blood transfusion rates (by ∼15%) in a number of surgical settings, the evidence remains weak for cardiac surgery patients despite the increased risk of anemia and related complications in cardiac vs. non-cardiac surgery patients [5,8,19,20,21,22,23]. In addition, preoperative IVIT studies in the setting of non-cardiac surgery have failed to demonstrate consistent improvements in other important clinical outcomes (length of hospital stay (LOS), infection and hospital readmissions) or morbidity and mortality, raising questions about the cost-effectiveness of IVIT [5,19,24,25].

In fact, the practice of limiting IVIT to the preoperative period is considered the main problem in the treatment of surgical patients with IDA, which is particularly important for those undergoing cardiac surgery, given the heightened risk of chronic anemia exacerbation and increased vulnerability to its complications after cardiac surgery [5].

Patients with cardiovascular diseases are increasingly frail but rarely represented in trials. Understanding effect modification by frailty on cardiovascular trials is critical as it could help define treatment strategies in frail patients. Frailty refers to a complex geriatric syndrome in certain individuals with decreased physiological reserves, resulting in increased vulnerability and susceptibility to stressors [26,27]. Like IDA, frailty is also more frequent in patients scheduled for cardiac surgery than in those undergoing elective non-cardiac surgery [28,29]. However, while frailty is considered an emerging concept in perioperative medicine as a risk factor for increased mortality, postoperative complications, prolonged LOS, poor functional recovery and readmission rates in a variety of major non-cardiac surgeries, it also remains poorly recognized and poorly investigated in patients undergoing cardiac surgery [28,29,30,31,32].

The concept of enhanced recovery after surgery (ERAS) has been practiced for decades and has been implemented in numerous surgical specialties. ERAS is a global surgical quality improvement initiative, and it is an element in the field of perioperative care. Several challenges and limitations exist in the implementation of ERAS that deserve consideration, including frailty, maximizing nutrition, prehabilitation, and treating preoperative anemia. Both frailty and correction of anemia are considered amongst the main challenges in the implementation of ERAS. However, ERAS has shown significant clinical outcomes, patient-reported satisfaction, and improvements in medical service cost. ERAS costs are higher than traditional care, but the patient’s clinical outcome and satisfaction are higher. ERAS has achieved significant benefits for patients and the health systems [33].

This study aimed to comparatively investigate the impact of postoperative IVIT as an add-on to preoperative IVIT on hemoglobin levels, allogeneic blood transfusion requirement, and clinical outcomes in frail versus non-frail patients with IDA in the setting of elective cardiac surgery.

## 2. Methods

### 2.1. Study Population

A total of 200 surgical patients with IDA (100 frail patients and 100 non-frail patients) who received IVIT before and/or after elective cardiac surgery (aortic valve replacement and/or coronary artery bypass graft surgery (CABG)) were included in this retrospective single-center study. The data was collected prospectively between January 2021 and November 2024. Patients were divided into four groups (*n* = 50 for each), based on frailty status and presence of add-on postoperative IVIT.

Group 1: frail pre/post group (frail patients receiving IVIT both in preoperative and postoperative period).

Group 2: non-frail pre/post group (non-frail patients receiving IVIT both in preoperative and postoperative period).

Group 3: frail pre group (frail patients receiving only preoperative IVIT).

**Figure 1:**
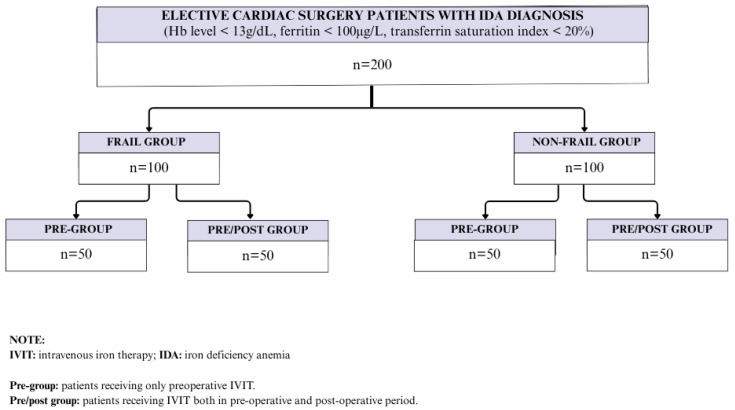
Flowchart of the study population.

Group 4: non-frail pre group (non-frail patients receiving only preoperative IVIT) The flowchart of the study population is illustrated in Figure 1.

The presence of contraindications for IVIT, undergoing emergent cardiac surgery, and presence of anemia related to renal dysfunction or infection were the exclusion criteria of the study.

Written informed consent was obtained from each subject following a detailed explanation of the objectives and protocol of the study which was conducted in accordance with the ethical principles stated in the “Declaration of Helsinki” and approved by the Bilkent City Hospital Clinical Research Ethics Committee (Protocol No: TABED-2-24-628).

### 2.2. Assessments

Data on patient demographics (age, gender), surgical intervention (type of operation, cardiopulmonary bypass (CBP) time, and aortic cross-clamp (X-clamp) time), perioperative parameters (amount of perioperative bleeding (mL), red blood cell (RBC) transfusion (U)), postoperative complications (reoperation for bleeding, surgical site infection), safety (adverse events related to IVIT), length of intensive care unit (ICU) stay and LOS were recorded in each group (Table 1) Postoperative follow-up parameters included change in hemoglobin levels and reticulocyte counts from baseline (recorded on operation day, postoperative day 1, day 7, 1st month and 3rd month) as well as the hospital readmission and mortality rates within 3 months after surgery are presented in Table 2.

### 2.3. Frailty Diagnosis

Frailty was identified using the Johns Hopkins Adjusted Clinical Groups (ACG) frailty-defining diagnosis indicators, which identify frailty by the presence of ≥1 diagnostic clusters based on 10 clusters of frailty-defining diagnoses. The Johns Hopkins Adjusted Clinical Groups (ACG) frailty-defining diagnosis indicators are presented in Table 3. (i.e., malnutrition, vision impairment, dementia, urinary incontinence, decubitus ulcer, loss of weight, poverty, fecal incontinence, difficulty in walking, and falls) [34].

### 2.4. IDA Diagnosis and IVIT Protocol

Preoperative/postoperative IDA was documented by hemoglobin levels < 13 g/dL, ferritin levels < 100 µg/L and transferrin saturation index < 20%. The IVIT before and/or after surgery was applied through a routine protocol which involves use of 1000 mg IV ferric carboxymaltose (i.v FCM; Inferject, Abdi İbrahim Pharma, İstanbul, Türkiye) injection.

### 2.5. Statistical Analysis

Statistical analysis was performed using IBM SPSS Statistics for Windows, Version 22.0 (IBM Corp., Armonk, NY, USA). The sample size was determined based on a similar study on the impact of postoperative IVIT. Using the protocol, 50 patients were deemed sufficient in each group to show a statistically significant difference with 5% error and 80% power (5).

The chi-square (χ^2^) test was used for the comparison of categorical data, while ANOVA and post hoc Tukey test were used for the parametric variables, applying Bonferroni correction for *p* values. Data are expressed as mean ± standard deviation (SD) and percent (%) where appropriate. *p* < 0.05 was considered statistically significant.

## 3. Results

### Patient Demographics, Surgical Intervention and Postoperative Outcome

No significant difference was noted between study groups in terms of patient demographics, surgical intervention, postoperative complications, length of ICU or hospital stay, hospital readmission or mortality rates (Table 1).

IVIT was not associated with a significantly increased risk of adverse events (relative risk: 4.50, 95% CI: 0.64–31.56).

Albeit not significant, there was a tendency for longer CBP time (92.0 ± 10.0 min) and X-clamp time (77.0 ± 8.0 min), higher amount of perioperative bleeding (715.0 ± 60.0 mL) and longer ICU stay (51.0 ± 10.0 h) in the frail pre group, when compared to other groups (Table 1). Also, groups without postoperative IVIT, regardless of baseline frailty, showed a nonsignificant tendency for higher volume of RBC transfusion (up to 2.6 U vs. up to 2.2 U) and higher rates for hospital readmission (8.0% and 6.0% vs. 4.0%) and mortality (2.0% vs. 0.0%) than those with postoperative IVIT (Table 1).

Changes in hemoglobin concentration and reticulocyte counts during 3-month follow-up: During 3-month follow-up, hemoglobin levels (from 10.6 ± 1.2 g/dL at baseline to 12.6 ± 1.4 g/dL at 1st month and to 13.4 ± 1.4 g/dL at 3rd month, *p* = 0.014 and *p* = 0.023, respectively) and reticulocyte counts (from 0.035 ± 0.005 × 10^12^/L at baseline to 0.075 ± 0.005 × 10^12^/L at 1st month and to 0.085 ± 0.005 × 10^12^/L at 3rd month, *p* = 0.0046 and *p* = 0.0021, respectively) were significantly improved from baseline only in the frail pre/post group (Table 2).

No significant improvement was noted in hemoglobin levels at postoperative 1st and 3rd months in the frail pre group, while reticulocyte counts in the postoperative 3rd month were also significantly higher than baseline values in this group (0.035 ± 0.005 vs. 0.065 ± 0.005 × 10^12^/L, *p* = 0.01) (Table 2).

Patients in the non-frail pre/post and non-frail pre groups showed no significant improvement in hemoglobin levels and reticulocyte counts throughout the postoperative follow-up (Table 2).

## 4. Discussion

Our findings indicate efficacy of postoperative IVIT in improving hemoglobin levels and reticulocyte counts starting from the first postoperative month after elective cardiac surgery in frail patients with IDA. Overall, use of postoperative IVIT in patients with IDA seems likely to reduce hospital readmission and mortality rates after elective cardiac surgery, regardless of the baseline frailty status. The prevalence of frailty is considered particularly high in cardiac surgery patients, as explained by the higher proportion of medically complex patients potentially presenting for cardiac versus non-cardiac surgery [28]. The high prevalence of frailty in cardiac surgery patients is important given that frail patients are more vulnerable to complexity of surgical process and more likely to experience procedural failure, postoperative complications, poor functional recovery and worsening frailty after hospitalization for CABG. Frailty screening for elderly patients objectively may have important aspects for risk reduction by taking preoperative precautions to increase the strength of the patients such as nutritional support, respiratory muscle reinforcement, exercise, and treatment of reversible comorbidities such as hypothyroidism, anemia, or depression [28,29,35]. The achievement of significantly improved hemoglobin levels and reticulocyte counts via postoperative IVIT only in our frail patients seems notable in this regard, as these patients were also found to be at increased risk of perioperative bleeding and RBC transfusion need, besides the prolonged CBP time and X-clamp time, if practice of IVIT was limited to the preoperative period. Concomitantly improved hemoglobin levels and reticulocyte counts from the first month of elective cardiac surgery in frail patients emphasize the efficacy of postoperative IVIT in inducing erythropoiesis besides the hemoglobin recovery in this group. However, the use of preoperative IVIT alone was effective in inducing erythropoiesis (increased reticulocyte counts) at the 3rd postoperative month but not hemoglobin recovery in the frail group.

Anemia, as a powerful prognostic factor for the development of frailty-related problems (i.e., muscle weakness, reduced performance, falls, and mortality), is considered likely to predispose or accelerate the development of frailty [36]. Hence, the frailty-specific efficacy of postoperative IVIT on improved hemoglobin and reticulocyte counts seems notable, since anemia in frail patients is suggested to be driven mainly by the systemic inflammatory response which is also responsible for the creation of a relative iron- and erythropoietin-deficient state postoperatively [25,37,38]. In addition, IDA has also been associated with a worsening of cardiac function, exercise capacity and quality of life (QoL) and with an increased risk for hospitalization and mortality, especially for frail patients [39,40]. In this regard, our findings emphasize the utilization of more inclusive risk scores (i.e., the Johns Hopkins ACG frailty indicator) in patients undergoing elective cardiac surgery to optimize correction of anemia and consideration of IDA as a modifiable risk factor that should be addressed before elective cardiac surgery and be treated pre/postoperatively, particularly in frail patients [28,31,41,42].

In patients undergoing major abdominal surgery, IVIT was reported to be associated with a significantly increased preoperative hemoglobin, fewer RBC transfusions and shorter LOS compared to non-treated anemic or non-treated IDA patients [43,44,45,46]. Available data on the use of IVIT for cardiac surgery patients are less robust compared with other surgical settings [5,8,21,22,47,48,49]. The analysis of 447 cardiac surgical patients revealed that 30% of IDA patients who received IVIT were restored to a non-anemic state before surgery and required fewer allogeneic blood transfusions compared to anemic patients [48]. Also, the use of IVIT before elective CABG surgery in 164 patients with IDA was associated with significantly reduced preoperative RBC transfusion rate, shorter LOS and lower in-hospital mortality, while a 1-unit decrease in preoperative hemoglobin level was found to be related to a 1.8-fold higher risk of mortality [49]. A total of 37,498 participants were studied and RBC transfusion was found as an independent factor of in-hospital mortality in isolated CABG surgery, even when a low volume of RBC transfusion was administered [50].

In patients undergoing on-pump cardiac surgery, use of preoperative IVIT (a single dose of 1000 mg ferric carboxymaltose) vs. placebo was reported to significantly reduce the need for RBC transfusions during the first 4 postoperative days (mean 0.3 vs. 1.6 red cell units) and to significantly increase the postoperative hemoglobin concentration at 4 days (mean 9.7 vs. 9.3 g/dL) and 6 weeks (mean 12.6 vs. 11.8 g/dL) after surgery [47]. Despite its statistical non-significance, the tendency for higher volume of RBC transfusion in our patients without postoperative IVIT than in those with postoperative IVIT (up to 2.6 U vs. up to 2.2 U) seems notable and clinically meaningful in this regard.

Currently, there is no substantial evidence in the literature to support the routine use of postoperative IVIT in cardiac surgery, while our results demonstrated that it might play a significant role in improving perioperative outcomes in the elective cardiac surgery setting especially for frail patients. In contrast, in a study with 120 patients with elective cardiac surgery (CPBG) and post-pump hemoglobin levels of 7–10 g/dL, postoperative IVIT used alone or in combination with low-dose erythropoietin was not found to be effective for correction of postoperative anemia, as evaluated at different time intervals until day 30 postoperatively [51]. Nonetheless, the postoperative period is suggested to be particularly favorable for implementing IVIT in terms of improved clinical outcomes after cardiac surgery in patients with IDA, given the direct effects of cardiac surgery on exacerbation of chronic anemia and increased physiological vulnerability to anemia-related complications [5]. The likelihood of postoperative IVIT to reduce hospital readmission and mortality rates after elective cardiac surgery, both in our frail and non-frail groups, is notable in this regard, emphasizing the considerable role of postoperative anemia in delayed postoperative patient recovery [16,25,37]. In addition, postoperative hospital stay and post-discharge follow-up appointments are considered likely to enable implementation of longer-term treatment with multiple sessions of IVIT to achieve a sustained therapeutic response [5]. Hence, there are ongoing studies specifically addressing the efficacy of postoperative IVIT in cardiac surgery patients with IDA, such as the POAM trial on clinical outcomes in patients with chronic IDA within 12 months of cardiac surgery [5] and the AGE ANEMIA study on the effect of postoperative IVIT on 90-day disability-free survival besides the change in reticulocyte hemoglobin content hemoglobin levels, hospital complications, dyspnea, QoL and functional outcomes in older cardiac surgery patients with postoperative IDA [8].

Certain limitations to this study should be considered. First, the potential lack of generalizability is an important limitation due to the single-center study design as well as the exclusion of patients with emergent cardiac surgery. Second, given the potential effects of IVIT on patient-centered outcomes, the lack of data on QoL, disability and functional outcomes is another limitation of the present study. Nevertheless, despite these limitations, our findings provide data on utilization of postoperative IVIT in IDA patients undergoing elective cardiac surgery in terms of clinical outcomes until the 3rd postoperative month and across the frailty subgroups.

In conclusion, our findings indicate the efficacy of postoperative IVIT in improving postoperative hemoglobin levels and reticulocyte counts starting from the first postoperative months, besides its potential in reducing perioperative RBC transfusions, in the setting of elective cardiac surgery in frail patients with IDA. IDA can be considered as a modifiable risk factor that should be addressed before elective cardiac surgery and be treated pre/postoperatively, particularly in frail patients. In this regard, utilization of more inclusive risk scores comprising the assessment of frailty status in patients with IDA undergoing elective cardiac surgery and not limiting the use of IVIT only to the preoperative period particularly in those with baseline frailty may optimize management of pre-existing state of acute-on-chronic anemia as well as its further exacerbation postoperatively. Further large-scale studies on certain high-risk patient groups are needed to justify the potential benefit of using postoperative IVIT in reducing the hospital readmission and mortality rates after elective cardiac surgery as well as to determine the best methods for improving patient outcomes and managing postoperative anemia in surgical patients with IDA.

## Figures and Tables

**Table 1:** Patient demographics, surgical intervention and postoperative outcome.

	Frail Pre/Post (*n* = 50)	Non-Frail Pre/Post (*n* = 50)	Frail Pre (*n* = 50)	Non-Frail Pre (*n* = 50)	*p* Value
**Patient demographics**					
Age (year), mean ± SD	77.0 ± 8.0	71.4 ± 7.0	75.1 ± 6.0	68.2 ± 6.0	>0.05
Gender, *n* (%)					
Female	36 (72.0)	31 (62.0)	33 (66.0)	38 (76.0)	>0.05
Male	14 (28.0)	19 (38.0)	17 (34.0)	12 (24.0)	
**Surgical intervention**					
Type of operation, *n* (%)					
CABG	39 (78.0)	41 (82.0)	37 (74.0)	42 (84.0)	>0.05
Valve replacement	6 (12.0)	6 (12.0)	8 (16.0)	5 (10.0)	
CABG + valve replacement	5 (10.0)	3 (6.0)	5 (10.0)	3 (6.0)	
CBP time (min), mean ± SD	89.0 ± 10.0	84.0 ± 8.0	92.0 ± 10.0	86.0 ± 8.0	>0.05
X-clamp time (min), mean ± SD	66.0 ± 7.0	69.0 ± 8.0	77.0 ± 8.0	71.0 ± 8.0	>0.05
**Perioperative parameters**					
Perioperative bleeding (mL), mean ± SD	655.0 ± 60.0	590.0 ± 55.0	715.0 ± 60.0	550.0 ± 50.0	>0.05
RBC transfusion (U), mean ± SD	2.0 ± 0.2	2.2 ± 0.3	2.6 ± 0.3	2.5 ± 0.3	>0.05
**Postoperative complications**					
Reoperation for bleeding, *n* (%)	2 (4.0)	3 (6.0)	3 (6.0)	2 (4.0)	>0.05
Surgical site infection, *n* (%)	1 (2.0)	2 (4.0)	1 (2.0)	0 (0.0)	>0.05
**Hospital outcome**					
Length of ICU stay (hours), mean ± SD	44.0 ± 10.0	45.0 ± 10.0	51.0 ± 10.0	46.0 ± 10.0	>0.05
Length of hospital stay (day), mean ± SD	5.8 ± 3.0	5.9 ± 3.0	6.1 ± 3.0	6.2 ± 3.0	>0.05
**Follow-up outcome**					
Hospital readmission, *n* (%)	2 (4.0)	2 (4.0)	4 (8.0)	3 (6.0)	>0.05
Mortality, *n* (%)	0 (0.0)	0 (0.0)	1 (2.0)	1 (2.0)	>0.05

Frail pre/post: frail patients with preoperative plus postoperative IV iron therapy; non-frail pre/post: non-frail patients with preoperative plus postoperative IV iron therapy; frail pre: frail patients receiving only preoperative IV iron therapy; non-frail pre: non-frail patients receiving only preoperative IV iron therapy; CABG: coronary artery bypass graft surgery; CBP: cardiopulmonary bypass; X-clamp: aortic cross-clamp; RBC: red blood cell, ICU: intensive care unit.

**Table 2:** Changes in hemoglobin concentration and reticulocyte count during 3-month follow-up.

	**Frail Pre/Post (*n* = 50)**	**Non-Frail Pre/Post (*n* = 50)**	**Frail Pre (*n* = 50)**	**Non-Frail Pre** (*n* = 50)
**Hemoglobin concentration** **(g/dL), mean ± SD**				
Baseline	10.6 ± 1.2	11.3 ± 1.3	10.8 ± 1.2	11.5 ± 1.2
Operation day	11.5 ± 1.3	12.1 ± 1.3	10.9 ± 1.3	11.9 ± 1.3
PO day 1	10.8 ± 1.1	11.0 ± 1.2	10.5 ± 1.1	11.1 ± 1.1
PO day 7	11.9 ± 1.4	12.5 ± 1.4	11.1 ± 1.3	12.3 ± 1.4
PO 1st month	12.6 ± 1.4 ^a^	12.8 ± 1.4	11.9 ± 1.4	12.5 ± 1.4
PO 3rd month	13.4 ± 1.4 ^b^	12.9 ± 1.4	12.2 ± 1.4	12.8 ± 1.4
**Reticulocyte count (×10^12^/L), mean ± SD**				
Baseline	0.035 ± 0.005	0.04 ± 0.005	0.035 ± 0.005	0.04 ± 0.005
Operation day	0.044 ± 0.005	0.043 ± 0.005	0.04 ± 0.005	0.045 ± 0.005
PO day 1	0.045 ± 0.005	0.04 ± 0.005	0.045 ± 0.005	0.05 ± 0.005
PO day 7	0.050 ± 0.005	0.055 ± 0.005	0.045 ± 0.005	0.05 ± 0.005
PO 1st month	0.075 ± 0.005 ^c^	0.055 ± 0.005	0.05 ± 0.005	0.055 ± 0.005
PO 3rd month	0.085 ± 0.005 ^d^	0.045 ± 0.005	0.065 ± 0.005 ^b^	0.05 ± 0.005

Frail pre/post: frail patients with preoperative plus postoperative IV iron therapy; non-frail pre/post: non-frail patients with preoperative plus postoperative IV iron therapy; frail pre: frail patients receiving only preoperative IV iron therapy; non-frail pre: non-frail patients receiving only preoperative IV iron therapy; PO: postoperative. ^a^ *p* = 0.023, ^b^ *p* = 0.014, ^c^ *p* = 0.0046 and ^d^ *p* = 0.0021; compared to baseline.

**Table 3:** The Johns Hopkins Adjusted Clinical Groups (ACG) frailty-defining diagnosis indicators.

Measurement Items	
**1. Malnutrition**	Nutritional marasmus Other severe protein–calorie malnutrition
**2. Dementia**	Senile dementia with delusional or depressive features Senile dementia with delirium
**3. Vision impairment**	Profound impairment, both eyes Better eye: moderate or severe impairment Lesser eye: profound
**4. Decubitus ulcer**	Decubitus ulcer
**5. Urinary incontinence**	Incontinence without sensory awareness Continuous leakage
**6. Loss of weight**	Abnormal loss of weight and underweight Feed difficulties and mismanagement
**7. Fecal incontinence**	Incontinence of feces
**8. Poverty and social support needs**	Lack of housing Inadequate housing Inadequate material resources
**9. Difficulty in walking**	Difficulty in walking Abnormality of gait
**10. Fall**	Fall on stairs or steps Fall from wheelchair

Patients defined as frail: ≥1 of the 10 items.

## Data Availability

The data that support the findings of this study are available from the corresponding author upon reasonable request.
